# Case 1/2017 - 26-Year-old Male with Rapidly Progressive Heart Failure

**DOI:** 10.5935/abc.20170018

**Published:** 2017-02

**Authors:** Laís Costa Marques, Rogério Silva de Paula, Ivna Lobo Camilo, Vera Demarchi Aiello

**Affiliations:** Instituto do Coração (InCor) HC-FMUSP, São Paulo, SP - Brazil

**Keywords:** Heart Failure, Cardiomyopathy, Dilated, Street Drugs, Atrial Flutter

The patient was a 26-year-old male, from the town of Medina, Minas Gerais state, coming from the city of Barueri, São Paulo state, hospitalized due to dyspnea and edema (April 19, 2013).

At the age of 24 years (July 18, 2011), he was referred to InCor complaining of dyspnea on heavy exertion for 2 months. Before that, he never had any cardiovascular symptom, and, after beginning specific medication, his clinical findings improved. The patient denied other cardiovascular symptoms, diabetes mellitus, arterial hypertension, dyslipidemia, smoking. He reported using illicit drugs (amphetamines and marijuana) and abusive alcohol consumption on weekends (20 beer cans). He reported prophylaxis for rheumatic fever with monthly use of benzathine penicillin from the age of 12 years to 17 years.

The clinical and laboratory assessments prior to referral revealed cardiopathy with ventricular dilatation.

His serology for Chagas disease was negative, and coronary angiography was normal. The echocardiogram revealed left ventricular systolic and diastolic diameters of 60 mm and 44 mm, respectively, and left ventricular ejection fraction of 51%. 

The physical examination on July 18, 2011, showed: weight, 99.7 kg; height, 1.70 m; body mass index, 31.14 kg/m^2^; heart rate, 60 bpm; blood pressure, 116/70 mm Hg; and normal pulmonary auscultation. Cardiac auscultation revealed the presence of third cardiac sound and systolic murmur (++/6+) over the mitral area, apex beat palpated on the precordium (left 5th intercostal space), displaced 2 cm from the left midclavicular line, with extension of 2 digital pulps. The examination of the abdomen and lower limbs was normal, and there was no jugular venous distention.

The electrocardiogram (ECG) on July 14, 2011, revealed atrial flutter with high-degree atrioventricular block, mean heart rate of 40 bpm, QRS duration of 100 ms, SÂQRS -30º, probable antero-superior divisional block (ASDB) and final conduction disorder (rsr') on V_1_ and V_2_ (Figure 1).

**Figure 1:**
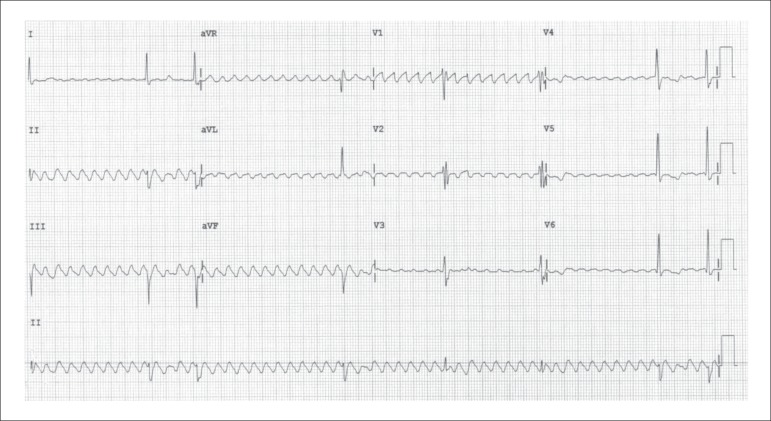
Electrocardiogram: atrial flutter with high-degree atrioventricular block, antero-superior divisional block, and right bundle-branch conduction disorder.

His chest X-ray showed pulmonary fields and hila, aorta and cardiac area within the normal range. 

The laboratory tests (July 14, 2011) revealed: hemoglobin, 17.9 g/dL; red blood cell count, 51%; leukocytes, 7640/mm^3^; creatinine, 1.3 mg/dL; sodium, 137 mEq/L; potassium, 4.7 mEq/L; total cholesterol, 189 mg/dL; HDL-C, 45 mg/dL; LDL-C, 117 mg/dL; triglycerides, 136 mg/dL; AST, 28 U/L; ALT, 44 U/L; TSH, 1.38 UI/mL; free T4, 1.03 ng/dL; TP(INR), 1.2; APTT(rel), 1.01; normal urinalysis; negative serology for Chagas disease.

The following drugs were prescribed: daily acetylsalicylic acid 300 mg, carvedilol 12.5 mg, losartan 25 mg, spironolactone 25 mg, and furosemide 40 mg. 

The new echocardiogram (Sept 2011) revealed left ventricular dimensions of 53x40 mm, ejection fraction of 48%, septal thickness and posterior wall of 11 mm, left atrial diameter of 34 mm, and diffuse left ventricular hypokinesia (Table1).

**Table 1:** Echocardiographic evolution

	September 2011	December 2012	April 2013
	41	44	45
Left atrium (mm)	34	47	50
RV (mm)	-	40	55
Ventricular septum (mm)	11	11	11
LV posterior wall (mm)	11	11	8
LVDD (mm)	53	56	66
LVSD (mm)	40	49	-
Ejection fraction (%)	48	27	20
Mass index (g/m^2^)	100	112	125
LV motility	Mild reduction	Marked reduction	Marked hypokinesia
RV motility	Mild reduction	Moderate reduction	Moderate hypokinesia
Mitral valve	Normal	Mild/moderate regurgitation	Moderate regurgitation
Tricuspid valve	Normal	Marked regurgitation	Marked regurgitation
Aortic valve	Normal	Normal	Normal
Right atrium	-	Enlarged	Enlarged

RV: right ventricle; LV: left ventricle; LVDD: left ventricular diastolic diameter; LVSD: left ventricular systolic diameter.

The 24-hour Holter showed persistent atrial fibrillation, with mean heart rate of 62 bpm, longest pause of 3.2s, 330 ventricular extrasystoles (14 VE/h), 1 paired extrasystole and 1 ventricular tachycardia with 3 beats.

Acetylsalicylic acid was replaced with warfarin, and electric cardioversion was programed 3 weeks after effective anticoagulation.

The first cardioversion was performed on December 13, 2011, with relapse of atrial fibrillation minutes after, and very low heart rate. 

The transesophageal echocardiogram (December 4, 2012) revealed: aorta, 44 mm; left atrium, 47 mm; ventricular septum and posterior wall, 11 mm; left ventricle (systole/diastole), 56/49 mm; ejection fraction, 27%; biatrial and biventricular enlargement, with moderate mitral and marked tricuspid valve regurgitation; aortic ectasia and no intracavitary thrombus (Table 1) (Figure 2).

**Figure 2:**
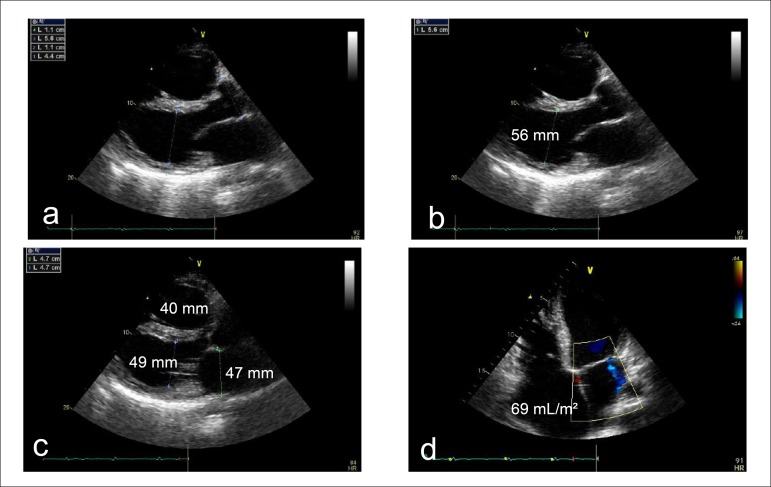
Transthoracic echocardiogram, longitudinal parasternal view (a, b, c) and four-chamber view (d) of the left ventricle. Note the enlarged atria and right ventricle.

New electric cardioversion was performed on the following day (December 5, 2012), with atrial fibrillation recurrence few minutes later. 

On April 19, 2013, the patient sought urgent medical care, reporting worsening of the dyspnea in the previous 4 months, with progression to occurrence at rest, orthopnea, abdominal volume enlargement and lower limb edema. In addition, he reported dry cough in the preceding week and marked worsening of dyspnea in the past two days.

The physical examination revealed: dyspnea; heart rate, 100 bpm; blood pressure, 100/80 mm Hg. The pulmonary auscultation evidenced rales on the bases. The cardiac auscultation revealed irregular heart rhythm, systolic murmur (+++/6+) over the mitral and tricuspid areas. The liver was palpated 3 cm from the right costal margin, and there was lower limb edema (++++/4+). 

The cough was attributed to heart failure (HF) because there was neither fever, nor leukocytosis nor images suggesting pneumonia on chest X-ray, which showed global cardiomegaly and a rectified middle arch (Figure 3). Intravenous furosemide and dobutamine, 5 *µ*g/kg/min, were administered.

**Figure 3:**
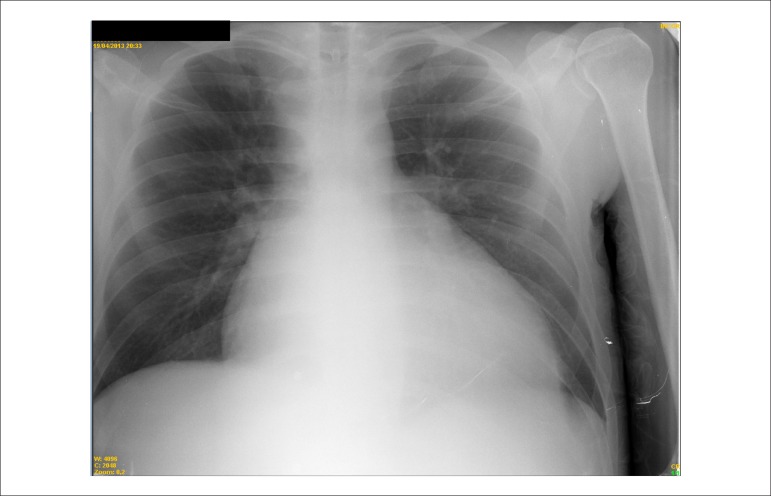
Chest X-ray (posteroanterior). Marked cardiomegaly, rectified middle arch and free pulmonary fields.

The laboratory tests (Table 2) evidenced kidney function deterioration and marked increase in the levels of brain natriuretic peptide (BNP) and C-reactive protein (CRP).

**Table 2:** Test results of the last admission

	April 19	April 23	April 26	April 27
Platelets/mm^3 ^	163000	135000	146000	22000
Red blood cell count (%)	51	44	44	53
Hemoglobin (g/dL)	16.4	14.4	14.2	16.2
Leukocytes/mm^3^	6280	6160	6910	10820
Neutrophils (%)	67	69	76	73
Segmented (%)	65	-	-	66
Cholesterol (mg/dL)	189	-	-	-
HDL-C (mg/dL)	45	-	-	-
LDL-C (mg/dL)	117	-	-	-
Triglycerides (mg/dL)	136	-	-	-
TSH (mIU/l)	-	3.79	-	-
Free T4 (µg/dL)	-	1.50	-	-
PT(INR)	2.6	2.4	2.0	
APTT (rel)	1.16	1.17	1.12	
Urea (mg/dL)	46	38	45	62
Creatinine (mg/dL)	1.33	1.60	1.60	3.07
GF (mL/min/1.73 m^2^)	69	56	56	26
Sodium (mEq/L)	140	141	138	143
Potassium (mEq/L)	4.2	4.0	3.7	5.2
AST (U/L)	86	-	539	2808
ALT (U/L)	84	-	205	983
Gamma GT (U/L)	93	-	-	104
APh (U/L)	57	-	-	69
Total bilirubin (mg/dL)	2.67	-	-	6.98
Direct bilirubin (mg/dL)	0.69	-	-	4.70
Total proteins (g/dL)	7.3	-	-	-
Albumin (g/dL)	3.5	-	-	-
Lactate (mg/dL)	-	62	-	122
BNP (pg/mL)	3540	2968	-	-
CRP (mg/L)	7.83	12.29	25.84	28.82
Arterial blood gas analysis				
pH	-	-	-	7.10
pO_2_ (mm Hg)	-	-	-	36.5
O_2_ saturation (%)	-	-	-	50
pCO_2_ (mm Hg)	-	-	-	43.6
HCO_3_ (mEq/L)	-	-	-	13.1
BE (mEq/L)	-	-	-	(-) 16.7

HDL: high-density lipoproteins; LDL: low-density lipoproteins; TSH: thyroid stimulating hormone; PT: prothrombin time; APTT: activated partial prothrombin time; GF: glomerular filtration; AST: aspartate aminotransferase; ALT: alanine aminotransferase; APh: alkaline phosphatase; BNP: brain natriuretic peptide; CRP: C-reactive protein; BE: base excess.

New electric cardioversion was indicated, as was a new transesophageal echocardiogram to rule intracavitary thrombi out.

The cough worsened, then with purulent sputum, and the association of tazobactam, piperacillin and azithromycin was introduced.

On April 26, 2013, the echocardiogram showed biatrial and biventricular enlargement, marked left ventricular and moderate right ventricular dysfunction, moderate mitral and marked tricuspid regurgitation, and no intracavitary thrombus (Table 1) (Figure 4).

**Figure 4:**
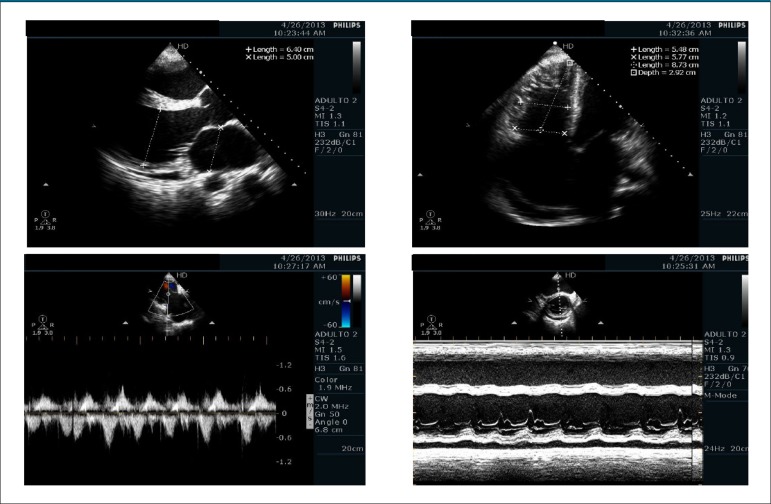
Transthoracic echocardiogram (April 2013): biventricular and biatrial enlargement.

Right after the exam, the patient had a decrease in his consciousness level and arterial hypotension, requiring orotracheal intubation for respiratory support and increased doses of vasoactive amines. 

On April 26, 2013, he had hyperthermia (38.6°C). Vancomycin was introduced, and the new chest X-ray was unaltered (Figure 5).

**Figure 5:**
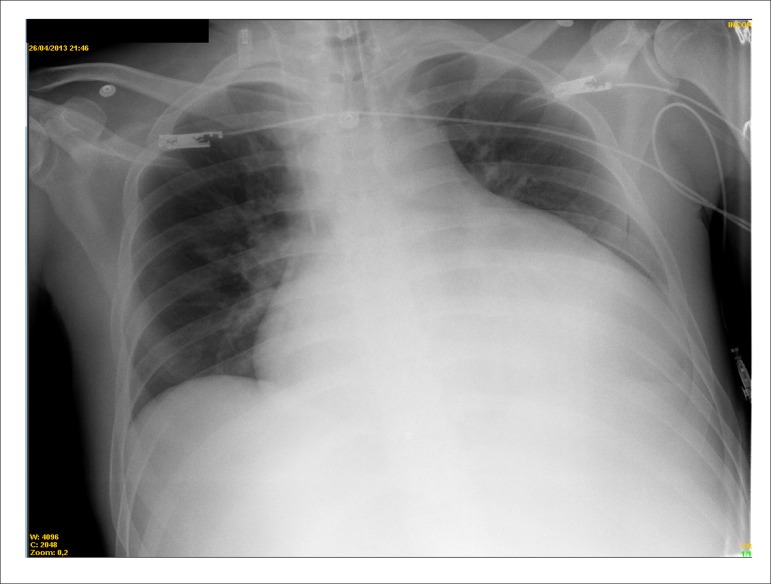
Chest X-ray (anteroposterior - bed). Marked cardiomegaly and free pulmonary fields.

Despite the administration of increasing doses of vasoactive amines, on April 27, 2013, the patient had shock and cardiac arrest, which was reversed. On the afternoon of that same day, he had hypotension and bradycardia, and an irreversible asystolic cardiac arrest.

## Clinical aspects

The patient was a 26-year-old male, reporting prophylaxis for rheumatic fever from the age of 12 years to 17 years, who, at the age of 24 years, developed dyspnea on heavy exertion, which improved with medication. However, after two years, dyspnea worsening and edema occurred (April 19, 2013). 

The clinical and laboratory assessments before the referral revealed cardiopathy with ventricular dilatation. The echocardiogram evidenced left ventricular systolic and diastolic diameters of 60 mm and 44 mm, respectively, and ejection fraction of 51%. His serology for Chagas disease was negative, and his coronary angiography, normal. On physical examination, a third cardiac sound and a systolic murmur (++/6+) over the mitral area were heard. The ECG showed atrial flutter, atrioventricular block with probable ASDB, and heart rate of 40 bpm. 

Over the following two years, the cardiopathy with ventricular dilatation evolved. Because the patient had a history of prophylaxis for rheumatic fever, rheumatic cardiopathy was considered as a possible etiology. However, the progressive and rapid course of our patient's illness is not commonly seen in patients without valvular damage consequent upon the acute event of rheumatic fever. The previous echocardiogram revealed left ventricular systolic and diastolic diameters of 60 mm and 44 mm, respectively, and ejection fraction of 51%. In September 2011, the patient showed: left ventricular dimensions of 53x40 mm; ejection fraction of 48%; septal and posterior wall thickness of 11 mm; left atrial diameter of 34 mm; and diffuse left ventricular hypokinesia without valvular damage.^1^ Therefore, other etiologies of dilated cardiomyopathy (DCMP) had to be considered for this case. 

Dilated cardiomyopathy is a progressive primary myocardial disease of unknown cause, characterized by a reduction in left ventricular or biventricular contractility.^2^ Approximately one in every three cases of congestive HF originates from DCMP.^3^ In addition to left ventricular or biventricular dilatation, it is characterized by contractile dysfunction, which results in congestive HF. Patients with DCMP have an increase in myocardial mass and in interstitial collagen,^4^ known as remodeling myocardial, which eventually leads to HF. Reversing that process to reduce morbidity and mortality remains a major challenge in health care practice.^5^ Our patient had echocardiographic changes compatible with DCMP and clinical findings of HF. On December 4, 2012, the transesophageal echocardiogram showed: aorta, 44 mm; left atrium, 47 mm; interventricular septum and posterior wall, 11 mm; left ventricle (systole/diastole), 56/49 mm; ejection fraction, 27%; biatrial and biventricular enlargement; and moderate mitral and marked tricuspid valvular regurgitation. On April 19, 2013, the patient sought medical care complaining of dyspnea worsening in the last 4 months, with progression to dyspnea at rest, orthopnea, increased abdominal volume and lower limb edema. The physical examination revealed congestive HF with pulmonary congestion, hepatomegaly, edema and bilateral atrioventricular valvular regurgitation.

Currently, DCMP accounts for around 10,000 deaths and 46,000 hospitalizations per year in the United States. In addition, DCMP is the major indication for cardiac transplantation.^6^ Although many cases lack an evident cause, DCMP either has a family origin or results from myocardial lesions produced by several known or unknown toxic, metabolic or infectious agents. It can be a late consequence of acute viral myocarditis, possibly partially mediated by immune mechanisms. It can occur at any age, being most often clinically apparent in the third or fourth decade of life. Reversible forms of DCMP may be found in cases of alcohol abuse, pregnancy, thyroid disease, cocaine use, and uncontrolled chronic tachycardia.^3^ The distribution of the DCMP causes is as follows: idiopathic, 50% of the cases; secondary to myocarditis, 9%; secondary to ischemic heart disease, 7%; consequent to infiltrative disease (amyloidosis and sarcoidosis), 5%; peripartum cardiomyopathy, 4%; secondary to systemic arterial hypertension, 4%; associated with human immunodeficiency virus (HIV) infection, 4%; post-connective tissue disease, 3%; substance abuse, 3%; doxorubicin use, 1%; and the other 10% comprise Chagas disease, Lyme disease, genetic causes, left ventricular non-compaction, and tachycardia-mediated cardiopathy.

Rheumatic fever remains the major cause of acquired cardiopathy in many regions, such as South America, Africa and India. It is frequently asymptomatic, especially rheumatic myocarditis. The most common clinical manifestations are arthritis and fever. Rheumatic fever and acute rheumatic myocarditis are under-represented in medical literature because they are rare in the United States and Europe.^7^

Although the first episode of acute rheumatic fever can lead to persistent valvular lesions, rheumatic cardiopathy most often results from cumulative valvular damage attributed to recurring acute rheumatic fever episodes, which can even be silent (no clinical symptoms). This makes its identification challenging. Rheumatic cardiopathy almost always affects the left-sided heart valves. Direct damage of right-sided heart valves is rare; they are usually affected as a result of the malfunction of the left-sided valves. In addition, narrowing of the mitral valve can develop, with blood flow obstruction, due to fusion of the leaflets or reduction in their mobility due to calcification.^8^ Left ventricular dilatation and HF have been mainly observed in patients with severe valvular heart disease. Although myocarditis is a common postmortem examination finding, the major cause of left ventricular dilatation and HF seems to be severe mitral regurgitation with or without aortic regurgitation.^9^ The ECG findings can include any degree of heart block, such as atrioventricular dissociation. The chest X-ray can show cardiomegaly. The echocardiography allows assessing the intensity of the valvular lesion, pericardial effusion, ventricular and atrial dilatation, and ventricular dysfunction.^10^ Therefore, rheumatic fever does not seem to be the most likely cause for this cardiopathy with dilatation and rapid and progressive aggravation. In addition, the clinical and image findings in this case are not those of rheumatic cardiopathy.

Sarcoidosis, another cause of cardiopathy with dilatation, can be considered in this case. Sarcoidosis is a granulomatous, non-caseous, heterogeneous disorder of unknown etiology, which can affect any organ. The heart involvement can be isolated or precede that of other organs (such as lung), or even occur simultaneously with that.^11^ The clinical manifestations of cardiac sarcoidosis depend on the location and extension of the granulomatous inflammation. Other cardiac manifestations comprise conduction disorders, ventricular and supraventricular arrhythmias, pericarditis and valvular dysfunction. In addition, the involvement of papillary muscles can lead to acute symptoms like those of hypertrophic cardiomyopathy with asymmetric septal hypertrophy, caused, however, by edema and not by hypertrophy of myocytes. The likelihood of heart disease caused by sarcoidosis should be considered for a healthy young or middle-aged individual with cardiac symptoms or a patient with known sarcoidosis who develop arrhythmias, conduction disorder or HF. That is an important etiology for our case, who had rapid and progressive worsening, arrhythmia, ventricular dilatation and clinical findings of HF, with no family history of genetic disease, and normal results of the other tests for heart disease.^12,13^

**(Rogério Silva de Paula, MD, and Ivna Lobo Camilo, MD)**

**Diagnostic hypothesis**: Heart failure secondary to cardiopathy due to sarcoidosis.

**(Rogério Silva de Paula, MD, and Ivna Lobo Camilo, MD)**

## Postmortem examination

**Figure 6:**
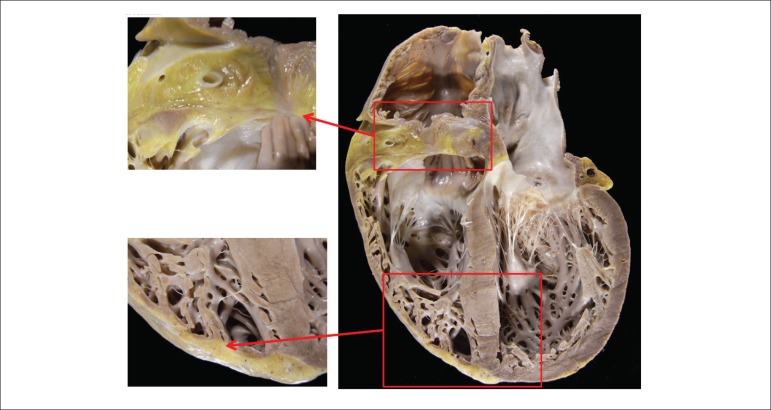
Gross aspect of the heart (four-chamber section). Significant fatty infiltration of the myocardium at the right ventricular base and apex, better evidenced in the magnifications (left panels). In addition, note global cardiomegaly with thinning of cardiac walls and biventricular dilatation.

**Figure 7:**
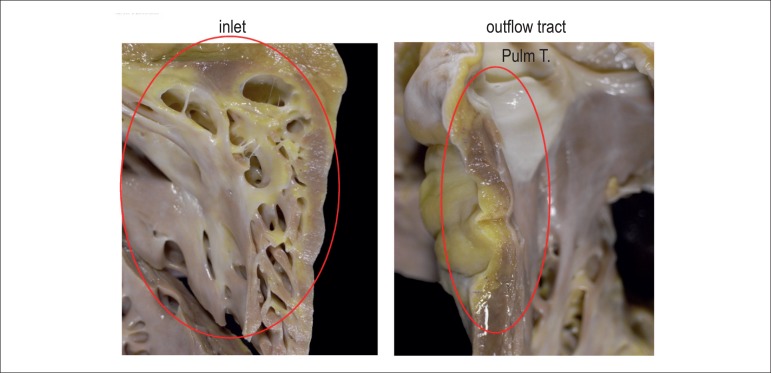
Gross aspect of the heart, longitudinal section of the right ventricular inlet and outflow tract. Both show focal fibrofatty replacement in the myocardial. Pulm T. - pulmonary trunk.

**Figure 8:**
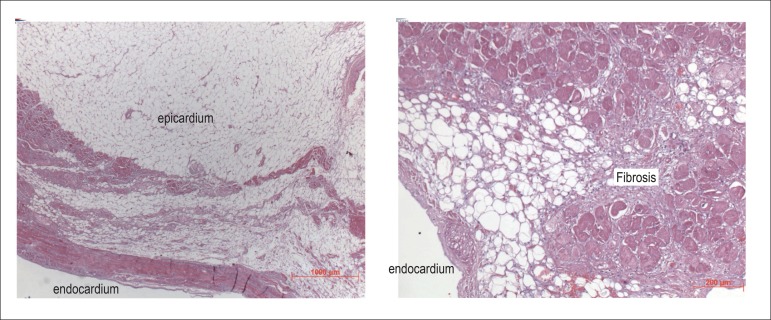
Microscopic sections of the right ventricular wall. Left panel: replacement of the muscle tissue with fibrofatty tissue. Right panel: the fibrotic component is better evidenced. Hematoxylin-Eosin objective magnifications, X 2.5 left and X 10 right, respectively.

**Figure 9:**
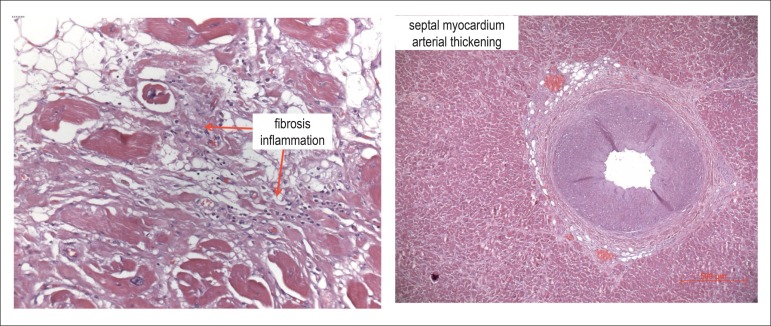
Microscopic sections of the right ventricular wall left panel and of the ventricular septum right panel. Left panel focal lympho-histiocytic inflammatory infiltrate and fibrosis between the cardiomyocytes. In addition to the fibrotic component, fatty tissue can be seen in the left upper corner of the image. Right panel arterial thickening in the septal myocardium due to hypertrophy of the tunica media. Hematoxylin-Eosin objective magnifications, X 20 left and X 5 right, respectively.

The heart weighed 680g and showed dilatation of the four chambers (Figure 6). The epicardial surface was smooth with sparse opaque whitish plaques. Its longitudinal section at the ventricular plane showed diffuse thinning of the ventricular walls and yellowish color due to focal adipose substitution in the right ventricular myocardium, mainly in the inlet, apex, diaphragmatic face and free wall of the subpulmonary infundibulum (Figures 6 and 7). There was no cavitary thrombus. The microscopic exam of the myocardium revealed, in addition to adipose infiltration of the right ventricle, focal fibrosis and lymphohistiocytic infiltrates, and signs of previous damage to cardiomyocytes (Figures 8 and 9). All ventricular walls showed hypertrophy of cardiomyocytes. The histological sections of the septal myocardium showed thickening of the wall and muscle arteries due to hypertrophy of the tunica media (Figure 9B).

The other organs revealed signs of chronic passive pulmonary and liver congestion due to congestive HF with terminal shock. In addition, there were thromboembolism of the small branches of the pulmonary bases, alveolar hemorrhage, serous ascites (3200 mL) and pericardial effusion (120 mL). Other signs of terminal heart failure included focal acute tubular necrosis, edema of renal tubular cells and cerebral edema. 

**Anatomopathological diagnosis**: Arrhythmogenic right ventricular cardiomyopathy (arrhythmogenic right ventricular dysplasia), congestive heart failure and morphological signs of terminal shock.

**Cause of death**: Cardiogenic shock 

**(Laís Costa Marques, medical student, and Vera Demarchi Aiello, MD)**

## Comments

The entity initially described as arrhythmogenic right ventricular "dysplasia" is currently known as arrhythmogenic right ventricular cardiomyopathy (ARVC), according to the European Society of Cardiology's position statement on cardiomyopathies.^14^

Its diagnosis is based on major and minor criteria, which comprise clinical, electrophysiological, hemodynamic and anatomopathological findings. 

In the case here described, the diagnosis of ARVC was not clinically established. From the anatomopathological viewpoint, however, both the gross and microscopic findings are typical, with adipose infiltration, and focal fibrosis and inflammation. Usually, the right ventricular involvement predominates, with little or no left ventricular involvement. In addition, global cardiomegaly is not usually found.

Phenotypic overlapping (global dilatation associated with fibrofatty replacement in the myocardium) might have hindered establishing the diagnosis during the patient's hospitalization.

The diagnostic criteria for ARVC established by a task force and published in 1994 were divided into major and minor. Those criteria include the presence of global and segment structural changes of the right ventricle, histological and ECG changes, arrhythmias and genetic factors. In 2010, a review of those criteria was published to help to identify ARVC and to diagnose it in the patients' family members.^15^ The diagnosis of ARVC requires the association of two major criteria, or one major criterion and two minor criteria, or even four minor criteria.

In the case here reported, in vivo endomyocardial biopsy was not performed, but ARVC was morphologically confirmed on postmortem examination, with the typical finding of myocardial areas of fibrofatty replacement in the right ventricular wall and of thickened arteries in the ventricular septum (previously described in this disease).^16^

Left ventricular involvement in ARVC has been reported in a study of 42 hearts from postmortem examination or from receptors of heart transplantation. That study has reported that approximately 50% of the specimens had gross involvement of the left ventricle, while 75% evidenced histological involvement. In addition to fibrofatty replacement in the myocardium in the sub-epicardial or middle-mural region, there was dilatation of that chamber in all cases with grossly evident disease, which was marked in 25% of them.^17^

## Pathogenesis and genetics

The ARVC consists in fibrofatty replacement in the myocardium. Myocardial atrophy is progressive and absent at birth. The myocardial process results from the death of cardiomyocytes beginning at birth.^18^ Postmortem studies have reported evidence of apoptosis in that cardiomyopathy.^19^ In addition, that same mechanism has been detected in biopsies (in vivo).^20^

The fibrofatty replacement occurs gradually from the epicardium towards the endocardium, becoming transmural. Consequently, there is weakening of the right ventricular free wall, causing dilatation and aneurysms, characteristically located between the inferior, apical and infundibular walls, forming the triangle of dysplasia.^18^

In addition to weakening the wall, those changes in association with the inflammatory factor hinder and delay the intraventricular electrical conduction, resulting in late potentials, epsilon wave and right bundle-branch block. Therefore, a ventricular arrhythmia can install due to reentry phenomenon.^18^

Two pathogenetic theories have been described.^21^ The first says the disease has a genetic component, and that the disorder in myocardial development begins in the intrauterine period.

Genetic studies^22^ have evidenced two types of inheritance for the ARVC phenotype. The first type is autosomal dominant inheritance with variable penetrance, while the second is represented by recessive forms associated with skin diseases. Ten genetic loci have been detected, but only five genes with mutations. The first ARVC-related gene was found in the Naxos disease, a rare recessive syndrome related to a mutation in the desmosomal protein called plakoglobin. That syndrome, however, has been characterized as the variant 2 of ARVC. The first form of autosomal dominant mutation in that variant was found in the gene that decodes the cardiac ryanodine receptor (RyR2), the receptor that accounts for calcium homeostasis and coordinates the excitation-contraction mechanism of cardiomyocytes. The mutations change the calcium-channel closing mechanism, and, thus, a high sympathetic stimulation via emotional or physical stress can increase excessively intracellular calcium, leading to severe arrhythmias. In addition to ARVC, mutations in the gene that decodes RyR2 can cause two other diseases: catecholaminergic polymorphic ventricular tachycardia and familial polymorphic ventricular tachycardia. The discovery of ARVC variant 2 is considered essential to unveil the pathogeneses of ARVC. Another recent discovery by the research team of Rampazzo^22^ has been in ARVC variant 1: the mutation of the genes that decode TGF-beta3. This cytokine stimulates the proliferation of mesenchymal cells. *In vitro* experiments have shown that the mutations in the genes that encode TGF-beta3 can cause myocardial fibrosis.

In addition, a theory speculates whether ARVC results from a previous infection (myocarditis, pericarditis). The theory considers a viral etiology that could be aggravated by an auto-immune reaction. The auto-immune or viral reaction could explain the inflammatory phenomenon, which would not occur due to only apoptosis of cardiomyocytes. That theory^21^ could explain left ventricular involvement and atrial rhythm disorders. The viral etiology has been suspected in a study^23^ with detection of viral genome in the myocardium of some patients with ARVC. Such theory, however, has been refuted in another study^24^ that advocates that the viruses are innocent bystanders, and that tissue degradation favors viral colonization.

Adipose infiltration by itself does not characterize ARVC, because some hearts have a certain amount of fat in the anterior and apical walls of the right ventricle and do not show degenerative changes in cardiomyocytes. Thus, to establish the diagnosis of ARVC one must identify the fibrous tissue replacement pattern associated with myocardial degeneration.^18^

Although ARVC is clinically recognized as a cause of cardiac sudden death in the young^25^ during physical exercise, there is a subgroup, in which our patient is included, with biventricular failure, unfavorable outcome and indication for heart transplantation. 

Cardiac magnetic resonance is useful to identify ARVC morphologically, and even to establish its prognosis.^26^

**(Laís Costa Marques, medical student, and Vera Demarchi Aiello, MD)**

**Section editor:** Alfredo José Mansur (ajmansur@incor.usp.br)

**Associated editors:** Desidério Favarato (dclfavarato@incor.usp.br)

Vera Demarchi Aiello (anpvera@incor.usp.br)
